# Monocyte‐to‐lymphocyte ratio as a predictor of stroke‐associated pneumonia: A retrospective study‐based investigation

**DOI:** 10.1002/brb3.2141

**Published:** 2021-05-04

**Authors:** Feng Cao, Yu Wan, Chunyan Lei, LianMei Zhong, HongTao Lei, Haimei Sun, Xing Zhong, YaDan Xiao

**Affiliations:** ^1^ Department of Neurology First Affiliated Hospital of Kunming Medical University Kunming China; ^2^ School of Public Health Kunming Medical University Kunming China

**Keywords:** acute stroke, infection, lymphocyte, monocyte, pneumonia

## Abstract

**Background and purpose:**

Early prediction of stroke‐associated pneumonia (SAP) is significant in clinical practice, as it is frequently challenging due to delays in typical clinical manifestations and radiological changes. The monocyte‐to‐lymphocyte ratio (MLR) has been proposed as an indicator of systemic inflammation and infection. However, none of these studies have focused on the predictive value of the MLR for SAP. We investigated the predictive value of MLR for SAP and investigated its relationship with disease severity.

**Methods:**

In this retrospective study, we assessed 399 consecutive patients with acute stroke. SAP was defined according to the modified Centers for Disease Control and Prevention criteria. The severity of pneumonia was rated using the pneumonia severity index (PSI). MLR was calculated by dividing absolute monocyte counts by absolute lymphocyte counts.

**Results:**

Among all the patients, SAP occurred in 116 patients (29.1%). White blood cell (WBC), neutrophil, monocyte, and MLR levels in the SAP group were higher than those in the non‐SAP group, while lymphocyte levels were lower (*p* < .05). Multivariable regression analysis revealed that the MLR (OR = 7.177; 95% CI = 1.190–43.292, *p* = .032) remained significant after adjusting for confounders. The ROC curve showed that the AUC value of MLR for SAP was 0.779, the optimal cutoff value of MLR for SAP was 0.388, with a specificity of 64.7% and sensitivity of 81.3%. The MLR levels were significantly higher in the severe pneumonia group when assessed by PSI (*p* = .024) than in the mild group. The AUC value of MLR was 0.622 (95% CI = 0.520–0.724, *p* = .024) in the severe pneumonia group. The optimal cutoff value of MLR was 0.750, with a specificity of 91.0% and a sensitivity of 33.0%.

**Conclusions:**

Our study shows that a high MLR is an independent risk factor for SAP and has a predictive value for severe pneumonia in patients with SAP.

## Graphical abstract

Monocyte to lymphocyte ratio (MLR) have been proposed as indicators of systemic inflammation and infection. However, none studies have focused on the predictive value of MLR for SAP. We investigated the predictive value of MLR for SAP and to investigate their relationship with disease severity.

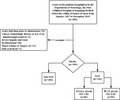

## 1 INTRODUCTION

Stroke is the leading cause of disability and death globally (Pinto et al., 2014). The high disability rate and mortality rate due to stroke are associated with infectious complications, the most common of which is pulmonary infection (Yuan et al., 2015). Stroke‐associated pneumonia (SAP) was first proposed by Professor Hilker in Germany in 2003 (Hilker et al., 2003). SAP is most common within 1 week after stroke, with an incidence of 7%**–**38% (Finlayson et al., 2011; Harms et al., 2013; Ji et al., 2013; Meisel & Smith, 2015; Smith, Kishore, Vail, et al., 2015; Sui & Zhang, 2011). It can significantly aggravate the condition of stroke patients, prolong the hospital stay, increase the cost of hospitalization, augment the incidence of severe disability, and increase the risk of death in stroke patients. (Smith, Bray, Hoffman et al., 2015). Early diagnosis and appropriate administration of antibiotics are essential for reducing SAP‐related morbidity and mortality (Hannawi et al., 2013; Meisel & Smith, 2015; Miller & Behrouz, 2016).

Various risk factors for SAP have been studied, including age, sex, smoking history, stroke severity, level of consciousness, dysphagia, feeding style, use of acid inhibitors, admission to the intensive care unit (ICU), history of hypertension, diabetes, chronic respiratory disease, and atrial fibrillation (Suda et al., 2018; Teh et al., 2018); however, their sensitivity and specificity for predicting SAP and the severity of pneumonia are variable and largely insufficient. Furthermore in the early stages, the clinical and radiographic features of SAP are often nonspecific, and sputum culture examination is time‐consuming, making it difficult to diagnose SAP (Kishore et al., 2015; Li et al., 2014; Smith, Bray, Hoffman et al., 2015; Warusevitane et al., 2016). Thus, a more objective and easily obtainable predictor is needed to assess the disease and simplify the diagnostic process. In recent years, research on the early prediction of SAP using simple and easily available blood parameters has gradually attracted attention (Nam et al., 2018; Zapata Arriaza et al., 2019).

The immune function reduction induced by stroke is an important internal mechanism of SAP, and the immunosuppression induced by stroke is mainly characterized by lymphocytosis and monocyte inactivation (Zuo et al., 2019). MLR, as a new immune inflammatory biomarker, has been proven to be useful as a predictor of systemic inflammation and infection, such as is caused by malaria, acquired immune deficiency syndrome (AIDS), active tuberculosis, and Guillen‐Barre syndrome (Huang, Ying, Quan et al., 2018; Naranbhai, Kim, Fletcher, & Cotton, 2014; Naranbhai, Hill, Karim, et al  2014; Warimwe et al., 2013). The diagnostic value of blood parameters in community‐acquired pneumonia (CAP), showing that MLR increased in patients with CAP and had predictive value for CAP (Huang, Liu, Liang et al., 2018). However, to our knowledge, no studies so far have investigated the relationship between MLR in the SAP and the association between MLR and the severity of pneumonia. Therefore, this study retrospectively investigated whether MLR, which is easily obtained from blood cell counts, is an independent predictor of SAP in patients with acute stroke.

## 2 MATERIALS AND METHODS

### 2.1 Patients selection

This study was conducted using retrospective data from the Department of Neurology, First Affiliated Hospital of Kunming Medical University. The overall registry project was approved by the hospital's authorities. For the present analysis, patients were included if they were admitted to the hospital between September 1, 2017, and December 31, 2019, within 24 hr of acute stroke.

Stroke patients who met the following criteria were included: (1) age ≥18 years; (2) all patients who were clinically diagnosed with stroke were principally admitted and registered at the time of discharge, after providing their informed consent. As initial evaluations for acute stroke, we conducted brain magnetic resonance imaging (MRI) or brain computerized tomography (CT) scans and laboratory examinations within the first 24 hr in all participants; (3) patients who had their blood parameters measured at admission. The exclusion criteria were as follows: (1) the presence of other conditions such as malignancy, hematologic disease, use of an immunosuppressant, active infection within the two weeks before admission, severe hepatic or renal dysfunction, major trauma, or surgery; and (2) missing blood parameter data at admission.

Patients with or without SAP within 1 week of onset of acute stroke were divided into SAP and non‐SAP groups, and the diagnostic criteria for SAP were based on the modified CDC criteria (Smith, Kishore, Vail et al., 2015). For patients with SAP, the PSI was used to evaluate the severity of SAP. Patients with SAP were divided into the severe group (PSI >90) and the mild group (PSI ≤90) (Nam et al., 2018).

### 2.2 Clinical characteristics and laboratory data collection

Baseline demographic and clinical covariates were collected at admission based on age, sex, and risk factors for stroke (hypertension, hyperlipidemia, diabetes mellitus, atrial fibrillation, stroke history, coronary heart disease, heart failure, chronic obstructive pulmonary disease, current smoking, and alcohol intake), indwelling gastric tube, acid drug treatment, mechanical ventilation, body mass index (BMI), ICU admission, hospital stay, and in‐hospital mortality. Initial stroke severity was assessed using the Glasgow coma score (GCS) and the National Institutes of Health Stroke Scale (NHISS) score. We used the quick sequential organ failure assessment (qSOFA) and Acute Physiology and Chronic Health Evaluation II (APACHE II) scores to assess the severity of the patient's condition. Laboratory examinations, including measurements of glucose profiles and blood parameters, were performed within 24 hr of admission. WBC, neutrophil absolute value count (neutrophil counts), monocyte absolute value count (monocyte counts), lymphocyte absolute value count (lymphocyte counts), and platelet count were recorded. MLR was calculated by dividing the monocyte count by the lymphocyte count. CT or MRI results of the head were also recorded to identify the type and location of the stroke.

Patients were diagnosed with SAP when they developed lower respiratory tract infections according to the modified CDC criteria (Smith, Kishore, Vail., 2015). The diagnosis of SAP was retrospectively conducted by two neurologists who were blinded to the other clinical and laboratory findings. To assess the burden or severity of SAP, we used the PSI score, which is a well‐validated scoring system for pneumonia burdens, in participants with SAP. (Porfyridis et al., 2014) For patients with SAP, PSI was further used to evaluate the severity of SAP. SAP patients were divided into severe (PSI >90) and mild (PSI ≤90) groups (Nam et al., 2018; Wang et al., 2019).

### 2.3 Statistical analysis

All statistical analyses were performed using the Statistical Package for the Social Sciences version 21.0 (SPSS). Normally distributed continuous variables are presented as the mean ±standard deviation, while other variables are presented as the median and interquartile range (IQR). Data were compared using the *t*‐test, Mann‐Whitney U‐test, chi‐squared test, or Fisher exact‐test, where appropriate. Variables that were identified as statistically significant by univariate analyses (*p* < .05) or clinically significant were entered into a multivariate logistic regression analysis to determine the contribution of MLR to SAP occurrence. All two‐tailed significance was set at *p* < .05. The AUC value, optimal cutoff value, sensitivity, and specificity were determined using ROC curves. AUC was compared using the DeLong test in the R version 4.0.3 package (https=//www.r‐project.org/).

## 3 RESULTS

### 3.1 Basic information of selected patients

**FIGURE 1:**
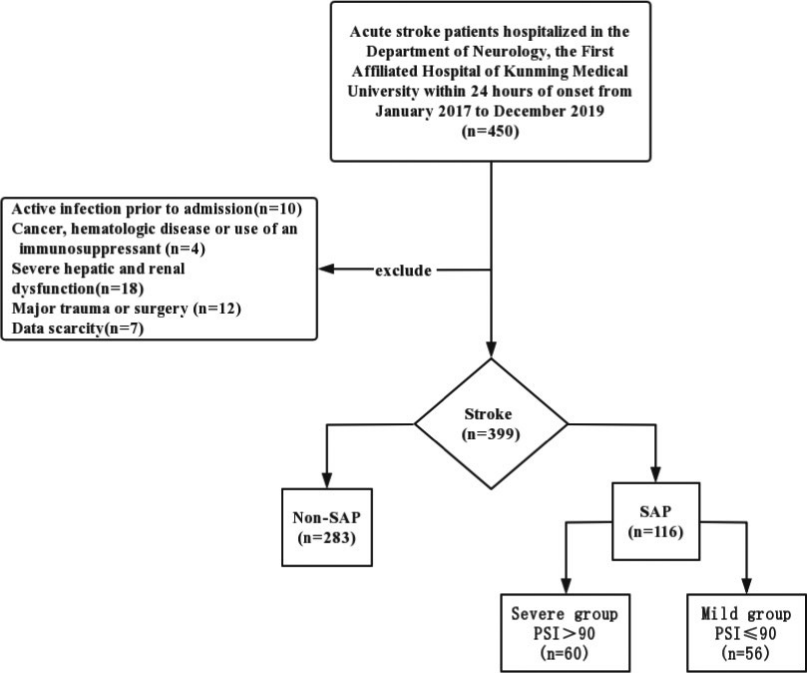
Flowchart of case selection. Flow diagram of the study recruitment Note: SAP, Stroke‐associated pneumonia; PSI, Pneumonia Severity Index

A total of 450 cases of acute stroke in the Department of Neurology, First Clinical Medical College of Kunming Medical University, from January 2017 to December 2019, were collected. After excluding 10 cases of infection, 4 cases of malignant tumor, 18 cases of severe hepatic and renal insufficiency, 7 cases of surgery, and 12 cases of incomplete data, 399 patients with acute stroke were finally included in the study. A total of 116 SAP cases (29.1%) were combined with 283 non‐SAP cases (70.9%). According to the PSI score of 90, SAP patients were divided into a severe pneumonia group (*n* = 60) and a mild pneumonia group (*n* = 56) (Figure 1).

Among the 116 patients in the SAP group, there were 70 males (60.3%), the mean age was 68.17 ± 12.96 years, 21 patients (18.1%) with cerebral hemorrhage, and 95 patients (81.9%) with cerebral infarction. Of the 283 patients in the non‐SAP group, 182 (64.3%) were male (64.3%), the mean age was (62.28 ± 13.27 years), cerebral hemorrhage occurred in 34 cases (12.0%), and cerebral infarction in 249 cases (88.0%). Patients with SAP were more likely to be older, with heart failure, indwelling gastric tube, using more acid suppression drugs, and being on mechanical ventilation than those in the non‐SAP group, patients with SAP were more severe at admission, as indicated by their higher initial NHISS scores and lower GCS scores, higher water swallow test scores, and lower BMI. WBC, neutrophil, and monocyte counts in the SAP group were higher than those in the non‐SAP group, whereas lymphocyte counts were lower. The median MLR value was 0.27[0.2‐0.37] in the non‐SAP group, which was significantly lower than that in the SAP group 0.45[0.33–0.68] (*p* < .05) (Table 1).

**TABLE 1:** Baseline characteristics of SAP and Non‐SAP patients

Variable	Non‐SAP (*n* = 283)	SAP (*n* = 116)	*p* value
Age, years, mean ± SD^a^	62.28 ± 13.27	68.27 ± 12.96	<.001
Sex (Male), *n* (%)^b^	182 (64.3)	70 (60.3)	.456
Hypertension, *n* (%)^b^	190 (67.1)	73 (62.9)	.421
Hyperlipemia, *n* (%)^b^	18 (6.4)	9 (7.8)	.614
Diabetes mellitus, *n* (%)^b^	53 (18.7)	22 (19.0)	.956
Atrial fibrillation, *n* (%)^b^	18 (6.4)	14 (12.1)	.057
Stroke history, *n* (%)^b^	53 (18.7)	23 (19.8)	.799
Coronary heart disease, *n* (%)^b^	27 (9.5)	10 (8.6)	.774
Myocardial infarction, *n* (%)^b^	5 (1.8)	2 (1.7)	.976
Heart failure, *n* (%)^b^	7 (2.5)	11 (9.5)	.002
COPD, *n* (%)^b^	5 (1.8)	6 (5.2)	.059
Current smoker, *n* (%)^b^	144 (50.9)	59 (50.9)	.997
Current drinker, *n* (%)^b^	133 (47.0)	56 (48.3)	.816
Stroke type	–	–	.109
Ischemic stroke, *n* (%)^b^	249 (88.0)	95 (81.9)	–
Hemorrhagic stroke, *n* (%)^b^	34 (12.0)	21 (18.1)	–
Location of stroke	–	–	.729
Subtentorial, *n* (%)^b^	58 (20.5)	22 (19.0)	–
Supratentorial, *n* (%)^b^	225 (79.5)	94 (81.0)	–
Gastric intubation, *n* (%)^b^	21 (7.4)	68 (58.6)	<.001
Acid suppression drugs, *n* (%)^b^	170 (60.1)	103 (88.8)	<.001
NHISS at admission, [IQR]^c^	3 [2–6]	11 [6–17]	<.001
GCS at admission, [IQR]^c^	15 [14–15]	11 [8–13.75]	<.001
Water swallow test, [IQR]^c^	1 [1–1]	1 [1–4]	<.001
WBC counts, (×10^9^/L)^a^	7.27 ± 2.05	9.76 ± 3.67	<.001
Neutrophil counts, (×10^9^/L)^a^	4.83 ± 1.96	7.78 ± 3.61	<.001
Lymphocyte counts, (×10^9^/L)^a^	1.79 ± 0.62	1.29 ± 0.62	<.001
Monocyte counts, (×10^9^/L)^a^	0.49 ± 0.17	0.60 ± 0.25	<.001
Platelet counts, (×10^9^/L)^a^	211.59 ± 62.31	194.19 ± 67.01	.014
MLR, [IQR]^c^	0.27 [0.20–0.37]	0.45 [0.33–0.68]	<.001
BMI (Kg/m^2^)^a^	24.08 ± 3.64	23.05 ± 3.62	.014
Mechanical ventilation, *n* (%)b	1 (0.4)	24 (20.7)	<.001

Abbreviations: BMI, body mass index; COPD, chronic obstructive pulmonary disease; GCS, Glasgow Coma Score; MLR, monocyte to lymphocyte ratio; NHISS, National Institutes of Health Stroke Scale; SAP, stroke‐associated pneumonia; WBC, white blood cell.

^a^ Mean±*SD*, *t‐test*.

^b^ *n* (%), chi‐square test.

^c^ [IQR], Mann–Whitney *U* test.

### 3.2 MLR have early predictive value in the SAP patients

A binary logistic regression analysis was performed to determine which factors could be suitable for predicting SAP. After further adjusting for confounding factors, the binary logistic analysis results showed that as the MLR increased the risk of SAP (OR = 7.177, 95% CI = 1.190–43.292, *p* = .032), neutrophil counts increased the risk of SAP (OR = 1.215, 95% CI = 1.060–1.393, *p* = .005). The risk of SAP increased with age (OR = 1.036, 95% CI = 1.090–1.063, *p* = .007), indwelling gastric tube (OR = 4.230, 95% CI = 1.906–9.387, *p* < .001), acid‐inhibiting drug use (OR = 2.216, 95% CI = 1.056–4.651, *p* = .035), mechanical ventilation use (OR = 4.137, 95% CI = (0.460–37.168), *p* = .205), and high NIHSS score on admission (OR = 1.107, 95% CI = 1.040–1.178, *p* = .001) were also risk factors for SAP in stroke patients (Table 2).

**TABLE 2:** Factors contributed to SAP by the multivariate analysis

Variables	OR	95%CI	*p*‐value
Age	1.036	(1.009–1.063)	.007
Subtentorial	1.244	(0.581–2.664)	.574
NIHSS	1.107	(1.040–1.178)	.001
BMI	0.965	(0.881–1.058)	.451
Gastric intubation	4.230	(1.906–9.387)	<.001
Acid suppression drugs	2.216	(1.056–4.657)	.035
Mechanical ventilation	4.137	(0.460–37.168)	.205
Water wallow test	1.200	(0.877–1.644)	.255
MLR	7.177	(1.190–43.292)	.032
Neutrophil counts	1.215	(1.060–1.393)	.005

Abbreviations: BMI, body mass indexMLR, monocyte to lymphocyte ratio; NIHSS, National Institutes of Health Stroke Scale; SAP, stroke‐associated pneumonia.

**FIGURE 2:**
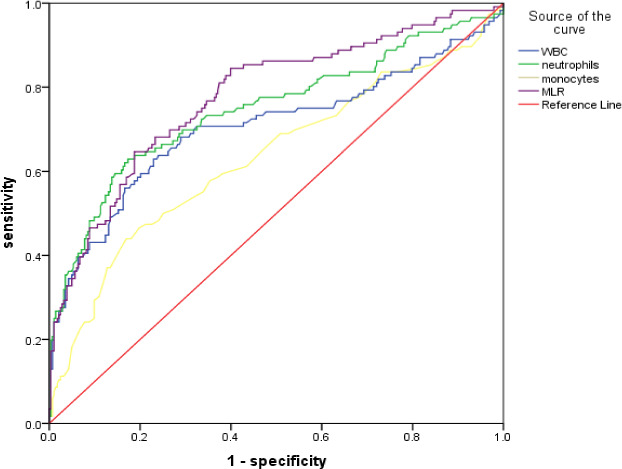
The ROC curve was used to evaluate the diagnostic value of blood parameters for SAP. ROC, receiver operating characteristic; SAP, stroke‐associated pneumonia; WBC, white blood cell; MLR, monocyte to lymphocyte ratio

The AUC in predicting SAP was 0.705 (95% CI = 0.640–0.770, *p* < .001) for WBC, 0.748 (95% CI = 0.689–0.807, *p* < .001) for neutrophils counts, 0.634 (95% CI = 0.569–0.698, *p* < .001) for monocytes counts, and 0.779 (95% CI = 0.726–0.831, *p* < .001) for MLR. No significant differences in the AUC were found (0.779 versus. 0.705, Z = 1.729, *p* = .084; 0.779 versus 0.748, D = 0.762, *p* = .446) for WBC and neutrophils, but MLR yielded higher AUC values than monocytes (0.779 versus 0.634, D = 3.401, *p* < .001). In addition, the optimal cutoff value of MLR for SAP was 0.388, with a specificity of 64.7% and a sensitivity of 81.3% (Figure 2 and Table 3).

**TABLE 3:** ROC curve was used to evaluate the diagnostic value of blood parameters for SAP

Variables	AUC	95%CI	*p* value	Optimal cutoff value	Specificity	Sensitivity
WBC	0.705	0.640–0.770	<.001	8.375	0.770	0.629
Neutrophil	0.748	0.689–0.807	<.001	6.605	0.827	0.629
Monocyte	0.634	0.569–0.698	<.001	0.645	0.830	0.506
MLR	0.779	0.726–0.831	<.001	0.388	0.647	0.813

Abbreviations: MLR, monocyte to lymphocyte ratio; SAP, stroke‐associated pneumonia; WBC, white blood cell.

### 3.3 The MLR was increased in the severe pneumonia group

**FIGURE 3:**
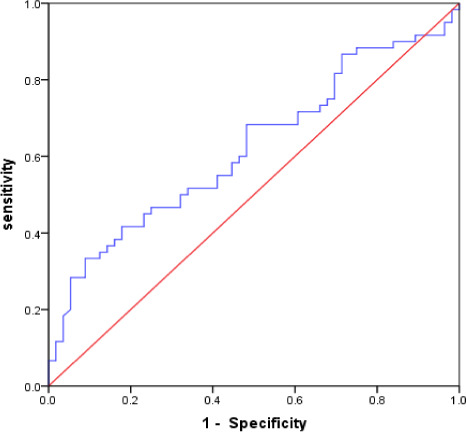
The ROC curve was used to evaluate the diagnostic value of MLR for SAP severity. ROC, Receiver Operating Characteristic; SAP, Stroke‐associated pneumonia; MLR, Monocyte to Lymphocyte Ratio

There were 116 patients with SAP; 60 SAP patients were in the severe group, while 56 were in the mild group. In the severe group, there were 43 males (71.7%), with a mean age of 74.55 ± 10.78 years, 11 patients (18.3%) with cerebral hemorrhage, and 49 patients (81.7%) with cerebral infarction; in the mild group, there were 27 males (48.2%) with a mean age of 61.55 ± 11.75 years, 10 patients (17.9%) with cerebral hemorrhage, and 46 patients (82.1%) with cerebral infarction. Compared with the SAP mild group, the SAP severe group had more patients of advanced age, of the male sex, who were smokers, and experienced atrial fibrillation, decreased GCS score and BMI, increased NIHSS score, qSOFA score, APACHE II score, and water swallow test score. The results showed that MLR in the SAP severe group was significantly higher than that in the SAP mild group (*p* < .05) (Table 4). The ROC curve was used to evaluate the predictive value of MLR in the severe pneumonia group. The results indicated that the AUC value of MLR was 0.622 (95% CI = 0.520–0.724, *p* = .024). The optimal cutoff value of MLR was 0.750, with a specificity of 91.0% and a sensitivity of 33.0%. (Figure 3).

**TABLE 4:** Baseline characteristics of patients with mild pneumonia and severe pneumonia groups

Variables	Mild group (*n* = 56)	Severe group (*n* = 60)	*p*‐value
Age, years, mean±SD^a^	61.55 ± 11.75	74.55 ± 10.78	<.001
Male, *n* (%)^b^	27 (48.2)	43 (71.7)	.010
Hypertension, *n* (%)^b^	31 (55.4)	42 (70.0)	.103
Hyperlipemia, *n* (%)^b^	5 (8.9)	4 (6.7)	.737
Diabetes mellitus, *n* (%)^b^	10 (17.9)	12 (20.0)	.769
Atrial fibrillation, *n* (%)^b^	3 (5.4)	11 (13.3)	.045
Stroke history, *n* (%)^b^	9 (16.4)	14 (23.3)	.351
Coronary heart disease, *n* (%)^b^	3 (5.4)	7 (11.7)	.325
Myocardial infarction, *n* (%)^b^	2 (3.6)	0 (0)	.231
Heart failure, *n* (%)^b^	3 (5.4)	8 (9.5)	.207
COPD, *n* (%)^b^	2 (3.6)	4 (6.7)	.680
Current smoker, *n* (%)^b^	23 (41.1)	36 (60.0)	.042
Current drinker, *n* (%)^b^	23 (41.1)	33 (55.0)	.134
Stroke type	–	–	.947
Ischemic stroke, *n* (%)^b^	46 (82.1)	49 (81.7)	–
Hemorrhage stroke, *n* (%)^b^	10 (17.9)	11 (18.3)	–
Location of stroke	–	–	.857
Subtentorial, *n* (%)^b^	11 (19.6)	11 (18.3)	
Supratentorial, *n* (%)^b^	44 (80.4)	49 (81.7)	
Gastric intubation, *n* (%)^b^	28 (50.0%)	40 (66.7%)	.069
Acid suppression drugs, *n* (%)^b^	49 (87.5%)	54 (90.0%)	.670
NHISS at admission [IQR]^c^	8 [3–14]	14 [9–18.75]	.001
GCS at admission [IQR]^c^	13 [9–15]	10 [7.25–13]	<.001
APACHE II at admission [IQR]^c^	8.5 [5–13]	13.5 [11–16]	<.001
QSOFA at admission [IQR]^c^	0 [0–1]	1 [0–1]	<.001
Water swallow test, [IQR]c	1 [1–3]	2 [1–4]	.048
WBC counts, (×10^9^/L)^a^	9.67 ± 4.07	9.85 ± 3.28	.792
Neutrophil counts, (×10^9^/L)^a^	7.67 ± 4.00	7.88 ± 3.24	.755
Lymphocyte counts, (×10^9^/L)^a^	1.35 ± 0.62	1.23 ± 0.62	.329
Monocyte counts, (×10^9^/L)^a^	0.57 ± 0.26	0.63 ± 0.24	.183
Platelet counts, (×10^9^/L)^a^	191.66 ± 55.58	196.56 ± 76.55	.695
MLR, [IQR]^c^	0.40 [0.31–0.56]	0.50 [0.34–0.87]	.024
BMI (Kg/m^2^)^a^	23.82 ± 3.62	22.32 ± 3.48	.025
Mechanical ventilation, *n* (%)^b^	10 (17.9)	14 (23.3)	.467
Hospital stay, days [IQR]^c^	12[9–16]	12[7.25–19]	.923
Hospital mortality, *n* (%)^b^	2 (3.6)	7 (11.7)	.099

Abbreviations: APACHE II, Acute Physiology and Chronic Health Evaluation II; BMI, body mass index; COPD, chronic obstructive pulmonary disease; CURB‐65, Confusion, Urea, Respiratory rate, Blood pressure, >65 years of age; GCS, Glasgow Coma Score; MLR, monocyte to lymphocyte ratio; NHISS, National Institutes of Health Stroke Scale; QSOFA, Quick Sequential Organ Failure Assessment; WBC, white blood cell.

^a^ Mean±*SD*, *t‐test*.

^b^ *n* (%), chi‐squared test.

^c^ [IQR], Mann–Whitney *U* test.

### 3.4 Outcomes of patients with or without SAP

Clinical outcomes between the groups with and without SAP are presented in Table 5. The SAP group showed more frequent ICU admission, longer durations of hospitalization, and a higher rate of in‐hospital mortality (*p* < .05).

**TABLE 5:** Outcomes of SAP and non‐SAP patients

Variable	Non‐SAP (*n* = 283)	SAP (*n* = 116)	*p*‐value
ICU admission *n* (%)	0 (0.0)	44 (37.9)	<.001
Hospital stay, days, [IQR]	10 [8–12]	12 [8–17]	<.001
Hospital mortality, *n* (%)	1 (0.4)	9 (7.8)	<.001

Abbreviations: ICU, Intensive Care Unit; SAP, Stroke‐associated pneumonia.

## 4 DISCUSSION

In this study, we found that a higher MLR was associated with SAP in patients with acute stroke and correlated with the severity of pneumonia. Furthermore, SAP significantly affected the clinical outcomes during hospitalization.

SAP was first proposed by Professor Hilker in Germany (Hilker et al., 2003), who stated that it could significantly aggravate the condition of stroke patients, prolong their hospital stay, increase the cost of hospitalization, increase the incidence of severe disability, and intensify the risk of death in stroke patients (Smith, Bray, Hoffman, et al., 2015). We found that 29.07% of patients in our study population had SAP, consistent with the idea that SAP is a common medical complication after stroke (Nam et al., 2018; Sacco et al., 2013). As in previous studies, our study indicated that the SAP group had worse clinical outcomes in terms of more lengthy hospitalizations and higher in‐hospital mortality rates (Nam et al., 2018). In our studies, there were several reasons for the worse clinical outcomes of patients with SAP. Patients in the SAP group had higher initial NHISS scores, lower GCS scores, and were older, so the patients in the SAP groups could have a more severe stroke and a higher risk of SAP, which accounts for the lower recovery rate (Nam et al., 2018; Westendorp et al., 2011). Stroke‐induced immunosuppression could also worsen the clinical outcomes of SAP (Liu et al., 2018). Fever, electrolyte imbalance, or hypoxia might have additional effects on the poor prognosis of these patients (Li et al., 2014; Nam et al., 2018; Vermeij et al., 2009).

As a new inflammatory and infectious biomarker, MLR has been proven to be applicable to the predictive and prognostic assessment of various inflammatory diseases such as malaria, AIDS, active tuberculosis, and Guillain‐Barre syndrome (Huang, Ying, Quan et al., 2018; Naranbhai, Hill, Karim et al., 2014; Warimwe et al., 2013). In 2018, Huang et al. found that MLR increased in patients with CAP, and MLR was positively correlated with C‐reactive protein (CRP) and procalcitonin (PCT), indicating that MLR had a predictive value for CAP (Huang, Liu, Liang, et al. 2018). Stroke‐induced immunosuppression syndrome (SIDS) is an important intrinsic mechanism of SAP that is mainly characterized by lymphocythemia and monocyte inactivation. MLR reflects the relationship between innate (monocyte) and adaptive (lymphocyte) immune responses, such as monocyte elevation and lymphocyte depletion. Therefore, the resulting increase in MLR is thought to have more power for predicting SAP than discrimination based on neutrophilia or lymphocytopenia alone (Zuo et al., 2019).

Howard & Simmons (1974) found that patients with acute stroke were susceptible to bacterial infection in the early stage and first proposed acquired immune deficiency after stroke (Howard & Simmons, 1974). Studies have shown that brain tissue damage and blood‐brain barrier damage after stroke, circulating immune cells like monocytes, neutrophils, and lymphocytes enter the brain tissue, leading to the autoimmune activation of the brain tissue that activates monocytes to cause more severe brain tissue damage in the brain. The body enters a state of immunosuppression to protect the damaged brain tissue, hence attenuating the autoimmune reaction in the brain. However, this stroke‐induced immunosuppression predisposes the body to pathogen invasion, leading to stroke‐induced immunosuppression syndrome (SIDS) and infection (Naess et al., 2017; Santos Samary et al., 2016).

To the best of our knowledge, no studies have reported the predictive value of MLR and SAP. In our study, we found that WBC, neutrophils, monocytes, and MLR levels in the SAP group were higher than those in the non‐SAP group, whereas the lymphocyte count and platelet count were lower. MLR yielded higher AUC values than monocytes. In addition, the optimal cutoff value of MLR for SAP was 0.388, with a specificity of 64.7% and a sensitivity of 81.3%.

For clinicians, early detection of severe SAP is very important, and active intervention measures could be taken early to improve the prognosis of patients. The 2019 consensus of Chinese experts on the prevention and treatment of stroke‐associated pneumonia indicates that the PSI score can be used to assess the severity of SAP patients (Wang, Lu, Xu et al., 2019). MLR was significantly correlated with the National Early Warning Score (NEWS), indicating the severity of *Klebsiella pneumoniae* infection, and MLR can help predict the severity of *Klebsiella pneumoniae* infection (Wang, Lu, Xu et al., 2019). In our study, the ROC curve for assessing SAP severity indicated that the AUC value of MLR was 0.622 (95% CI = 0.520–0.724, *p* = .024). The optimal cutoff value of MLR was 0.750, with a specificity of 91.0% and a sensitivity of 33.0%. Our studies also indicated that MLR had a predictive value for the severity of SAP. However, the mechanism of the association between higher levels of MLR and the severity of SAP is not yet clear.

Our study had certain limitations. First, this was a single‐center retrospective study with a relatively limited sample size. Second, our study did not include inflammatory indicators such as CRP and PCT, as well as risk factors for stroke‐related pneumonia, nutritional status, and oral function, which could have an impact on our conclusions. Third, we only used spot parameters for the analysis, and no follow‐up values were available. Therefore, further prospective studies comprising a greater number of patients from different centers are needed to validate the clinical value of MLR for SAP.

## 5 CONCLUSION

In conclusion, our study is the first to report the relationship between early prediction and severity of MLR and SAP. We suggest that MLR, a widely used and inexpensive inflammatory biomarker, could be useful in the early prediction of SAP occurrence and severity.

## CONFLICT OF INTERESTS

The authors declare that there are no conflicts of interest associated with this study.

## AUTHORS' CONTRIBUTIONS

Feng Cao was responsible for collecting data from patients with SAP, performing a literature search, statistical evaluation of the data, and writing the manuscript. Yu Wan collected data from patients with stroke and document retrieval, while ChunYan Lei and LianMei Zhong were involved in writing and correcting the manuscript. HongTao Lei performed statistical analysis. HaiMei Sun participated in the literature search, manuscript writing. Xin Zhong and YaDan Xiao collected data from stroke patients.

## ETHICAL APPROVAL

The study was conducted in accordance with the Declaration of Helsinki of the World Medical Association. Given that this is a retrospective study, the requirement for ethical approval was waived.

### PEER REVIEW

The peer review history for this article is available at https://publons.com/publon/10.1002/brb3.2141.

## Data Availability

The raw data supporting the conclusions of this manuscript will be made available by the author Haimei Sun ( haimeisun@139.com ), without undue reservation, to any qualified researcher.
